# Bullying Experiences of South Korean Nursing Students During Clinical Practice: A Focus Group Study on Two Colleges

**DOI:** 10.3390/healthcare14091182

**Published:** 2026-04-28

**Authors:** Misook Park, Chung-uk Oh

**Affiliations:** 1 Department of Nursing, Korea National University of Transportation, 61 Daehak-ro, Jeungpyeong-gun 27909, Chungcheongbuk-do, Republic of Korea; mspark@ut.ac.kr; 2 Department of Nursing, Kangwon National University, 346 Hwangjo-gil, Samcheok-si 24341, Gangwon-do, Republic of Korea

**Keywords:** bullying, descriptive phenomenology, focus groups, nursing, students

## Abstract

**Background/Objectives**: Nursing students are often exposed to bullying in clinical settings. Bullying in a practice environment is an important issue that causes psychological, physical, and mental health problems in nursing students. However, in South Korea, few qualitative studies have examined bullying among nursing students in clinical practice environments. This study aimed to explore the lived experiences of bullying among South Korean nursing students during clinical practice. **Methods**: A qualitative descriptive study using a semi-structured interview guide was employed to collect data from nursing students in a focus group. Overall, three focus groups were used, with seven students in each group comprising males and females. The sample size was based on data saturation and saturated on three focus group discussions, giving a sample size of 21. Purposeful sampling was used to select students who had completed one or more semesters of clinical practice (six credits, 270 h) from two nursing colleges. Interviews were conducted in May 2025. Data were analyzed using Colaizzi’s method. **Results**: A total of 23 meaning units, 14 themes, and 7 thematic clusters were identified. Seven overarching thematic clusters emerged: (1) exposure to harsh speech; (2) experiencing physical harm; (3) being considered a sexual object; (4) disrespected as a nursing student; (5) assigned tasks beyond an individual’s capabilities; (6) restricted educational opportunities corresponding to clinical practice objectives; and (7) discriminatory treatment due to healthcare professionals’ prejudice. **Conclusions**: These findings highlight the need for raising the awareness of bullying and applying the strategies to prevent bullying and protect nursing students during clinical practice.

## 1. Introduction

Bullying is a persistent phenomenon in nursing [1]. The expression of “Nurses eat their young?” [2] starkly illustrates the severity of workplace bullying among nurses. In South Korea, the term *Taewom*, which means “to burn someone down until they turn to ashes,” is also used to describe the phenomenon of workplace bullying [3].

Bullying is defined as the repeated exhibition of negative behaviors by the perpetrator toward the target, manifesting as verbal, physical, or psychological abuse in both overt and covert forms, and involving intentionality and a power imbalance [1]. Workplace bullying, incivility, and violence related to bullying are described in the literature as follows. Workplace bullying (WPB) is defined as “incidents where staff are abused, threatened or assaulted in circumstances related to their work, including commuting to and from work, involving and explicit or implicit challenge to their safety, wellbeing or health” [4]. Bullying typically involves repeated negative acts within a power imbalance, whereas incivility refers to low-intensity disrespectful behaviors [5]. Meanwhile, incivility is considered less threatening than bullying. Incivility, prominent and widespread [5], is described as low-intensity deviant behavior with ambiguous intent to harm the target, in violation of workplace norms for mutual respect. Uncivil behaviors are characteristically rude and discourteous, displaying a lack of regard for others [6]; uncivil actions such as neglect, scolding, harsh language, belittling remarks, sarcasm, gossiping, harassment, physical attacks, and sexual harassment negatively affect mental health [7] and cause physical stress [8]. Although studies on bullying and its attributes have been ongoing [1,5], the term “bullying” is sometimes used interchangeably with the words horizontal and vertical violence, and more recently incivility [9]. Previous research has indicated that there may be obvious overlaps between categories in the existing taxonomies [10].

Unfortunately, bullying is not a problem limited to nurses alone. Being bullied is a common experience for nursing students [1]. In fact, research indicates that nurses often first witness or experience workplace bullying not during their early careers as new nurses, but rather during their time as student nurses in training [11]. Nursing students are particularly vulnerable to bullying due to the inherent challenges of nursing, unfamiliar clinical environments, low reporting rates of bullying incidents, and limited clinical experience [12,13]. Nursing students are more prone to bullying from clinical instructors and registered nurses during clinical practice than on campus [14].

Clinical practice education involves on-site training that offers opportunities to apply theoretical knowledge to nursing practice, engage in communication with patients and other healthcare professionals, and acquire the knowledge and skills necessary to manage the complexity, uncertainty, and conflicts encountered in real-world practice [15]. In Korean nursing education, nursing students must complete clinical practicums to earn at least 22 credits in clinical practice courses, in addition to liberal arts and major theory courses, enabling them to develop the competencies required of professional nurses [16]. A previous meta-analysis including 28 studies (N = 9511) from 13 countries reported a global pooled prevalence of bullying among nursing students during clinical practice of 65.6% (95% CI 55.75 to 74.27). Among the subgroups, South Korean nursing students have reported a notably high prevalence of 87.22% (95% CI 78.40 to 92.77) [17]. Experiencing bullying during clinical practice increases stress, depression, and academic burnout among nursing students, while decreasing their clinical performance, self-esteem, and self-efficacy. It also adversely affects their career identity, satisfaction with their major, and professional self-image [18,19,20,21,22,23]. Bullying is often considered an unavoidable aspect of clinical practice settings [1]. Furthermore, bullying is frequently perceived as an ingrained part of nursing culture within healthcare settings [11,24,25]. Students may internalize this culture of bullying and, upon attaining positions of authority, perpetuate it, thereby creating a repetitive vicious cycle [26].

Previous studies on bullying experienced by nursing students during clinical practice include various reports, such as qualitative research on violence and bullying among nursing students conducted overseas [14,19,27,28,29,30,31,32]. However, because South Korea’s systems and culture differ from those of other countries, it is challenging to directly apply foreign research findings to the South Korean context. In South Korea, prior research has primarily focused on quantitative studies of bullying experienced by nursing students during clinical practice [11,18,20,21,22,23]. Qualitative research [23,33] exploring bullying among Korean nursing students remains limited. Therefore, qualitative research is needed to explore how nursing students interpret and experience bullying within Korean clinical training environments. Considering the negative impact and severity of bullying, a qualitative study is necessary to provide a comprehensive and in-depth exploration of the meaning of bullying experienced by nursing students in clinical practice settings in South Korea.

Phenomenology is a research approach that explores phenomena from the perspective of the individuals experiencing them, aiming to reveal the essence of these phenomena [34]. Colaizzi’s phenomenological research method is a technique used to describe and understand phenomena related to a specific topic, particularly when there is limited knowledge about the phenomenon under investigation. This approach has strengths in deriving deep understanding through a systematic, step-by-step analytical process [35]. The research topic of this study is, “What are the experiences of bullying encountered by nursing students during clinical practice?” Accordingly, this study aimed to explore the lived experiences and meanings of bullying among South Korean nursing students during clinical practice by applying Colaizzi’s phenomenological analysis through focus group interviews.

## 2. Materials and Methods

### 2.1. Study Design

A qualitative descriptive design using focus groups was employed. This study used phenomenological analysis based on Colaizzi’s methodology [35], intending to gain a comprehensive understanding of the bullying experienced by South Korean nursing students during clinical practice.

This study aimed to efficiently gather diverse perspectives by conducting focus group interviews in which multiple participants listened to one another, exchanged immediate feedback, and clarified their own viewpoints.

Focus groups in phenomenology-informed studies allow interviewees to elaborate on and share issues raised [36]. The use of focus groups serves as a means to enhance the credibility of the research by providing an environment that promotes interaction among participants and clarification of dialog [37], thereby offering a deeper understanding of the phenomenon under study [38].

### 2.2. Participants

The focus group interviews involved nursing students enrolled at two nursing colleges in South Korea. The eligibility criteria required participants to have completed six or more clinical practice credits and more than 270 practice hours since students with this level of experience are better equipped to provide comprehensive accounts of their varied clinical encounters. Recruitment began with an Institutional Review Board (IRB)-approved announcement posted on online student bulletin boards, targeting nursing students and inviting them to take part voluntarily. Participants were recruited using purposeful sampling to ensure that they had experience in clinical practice. Snowball sampling was used as a supplementary strategy when additional participants were needed. Because relying on socially close connections might limit the diversity of opinions, interviews were conducted in comfortable, quiet, and private settings with individual partitions to facilitate open sharing of personal views. Participants were interviewed in soundproof seminar rooms at a library as well as in separated private rooms within a café. To facilitate effective interactions during the sessions, participants from the same college were grouped. Both schools are four-year national universities with an annual enrollment of no more than 60 students per grade, making them comparable. At both institutions, the clinical practice takes place in hospitals with over 300 beds, community health center, center for mental health, nursing home, etc., where students rotate through different wards in two-week blocks. However, the two schools differ in the number of clinical practice sites and total practice hours. To capture the experiences of both first-time and repeat clinical practice students across various practice sites, participants were selected from two different regions and two academic years with differing clinical credit requirements. Two focus groups, each consisting of fourth-year students from a university in the Chungbuk region, were interviewed; these students had completed practices in adult, pediatric, psychiatric, and community nursing. Additionally, one focus group of third-year students from a university in the Gangwon region was interviewed; these participants had completed clinical practices in adult, pediatric, and maternal nursing.

In this study, the number of participants in each focus group was set at seven for the interaction benefit of the group, based on previous research [39,40,41]. One focus group was established within the nursing department of a college in the Gangwon region, and two focus groups were formed within the nursing department of a college in the Chungbuk region, yielding three focus groups in total. Focus group interviews foster empathy and reflection on the topic through mutual respect by listening to the diverse voices of others. However, when the topic is private or sensitive, it can cause stress for participants and may require strict confidentiality measures [35]. The study involved 21 participants, including seven third-year students (33.3%) and 14 fourth-year students (66.7%), ranging in age from 21 to 38 years with a mean age of 23.2 years. Regarding clinical experience, 7 students (33.3%) had completed one semester, while 14 students (66.7%) had completed three semesters. The sample comprised 18 females (85.7%) and 3 males (1 in Group 1, 2 in Group 3) (14.3%). Data saturation was considered achieved when there was repetition of themes or an absence of new emerging codes during the analysis of the third focus group. There were no eliminations among the participants.

### 2.3. Data Collection

On 9, 28, and 30 May 2025, three focus groups interviews were conducted face to face in seminar rooms or separated rooms of cafés affiliated with the participants’ educational institutions. Focus group interviews involve assembling individuals into groups to facilitate in-depth discussion. This format encourages interactions not only between researchers and participants but also among group members, generating synergistic effects that enable the efficient collection of rich and diverse data on predetermined topics within a limited timeframe [36]. Each focus group consisted of seven nursing students, with one researcher present during each session. All moderators were female registered nurses with PhDs and prior experience in qualitative research, who oversaw the focus groups had demonstrated proficiency in conducting focus group interviews, and possessed extensive knowledge of the subject matter. One researcher conducted interviews with a single focus group, while another interviewed two focus groups. Since participants might suppress their expressions through self-censorship due to concerns about potential disadvantages arising from sharing sensitive interview content within the group, the interviewer was designated as a researcher affiliated with an organization outside the interview group. This arrangement was designed to prevent any conflicts of interest between the researchers and the participants. Their duties included managing the timing of each session, taking detailed field notes, and fostering a comfortable environment. To facilitate interviews and encourage attentive listening, participants with dominant voices were allotted limited interview time, while those who were less talkative or more reserved were given more opportunities to speak. This approach fostered an environment where everyone could participate equally. To protect participants’ privacy, an explicit confidentiality agreement was established within the group, and all data were anonymized. Only the researchers had access to the research information, and all research materials were destroyed upon completion of the study. Participants were informed that any records mentioning specific individuals, events, or places could be deleted or modified. Each group’s interview was conducted once and was not repeated. Prior to the interviews, the moderators documented any pre-existing assumptions related to the phenomenon under study and actively sought to minimize subjective bias. During the interviews, the moderators deliberately avoided asking leading questions or implicitly steering participants toward specific responses, instead encouraging spontaneous and authentic expression. The interviews were scheduled at times and locations chosen by the participants, either on or off campus, to minimize interruptions and create a comfortable setting. Rapport was established through informal dialog at the outset, accompanied by a brief self-introduction to collect preliminary demographic data before starting the formal interview. The interview protocol comprised open-ended, semi-structured questions (Table 1). Interview protocol was tested at two induction meetings and using one pilot interview. After obtaining the participants’ informed consent, the interviews were audio-recorded using the Clovanote app (PC version) to ensure accurate data capture. Field notes were systematically recorded during the sessions to document the participants’ non-verbal behaviors (e.g., movements, facial expressions, vocal tone) and other relevant observations.

### 2.4. Data Analysis

The interviews were documented using the Clovanote app, which provides automatic Korean language transcription to convert audio into text. The transcription data was anonymized, the post-transcription data was transferred to an encrypted drive, and the data was deleted from the transcription drive to ensure the confidentiality of the research data. The moderator facilitating the interviews thoroughly reviewed the transcripts to correct typographical errors. Subsequently, a core researcher performed additional verification of the transcripts. The final transcripts consisted of 62 pages of A4-sized sheets.

Colaizzi’s seven-stage phenomenological analysis method [35] was used to analyze the qualitative data obtained from the interviews. These seven stages were as follows:

1. Transcription of Participant Interviews: All interviews were transcribed verbatim. The researchers independently and repeatedly read the transcripts to gain a deep understanding of the data.
2. Identification of Significant Statements: A total of 177 meaningful and relevant statements related to the phenomenon were extracted from the transcripts.
3. Reorganization and Generalization of Significant Statements: The selected statements were rephrased without altering their essence and were expressed in more general terms.
4. Formulation of Meanings: The researchers interpreted and analyzed the underlying 23 meanings conveyed in the participants’ statements.
5. Development of Thematic clusters and Themes: The formulated meanings were grouped into 7 thematic clusters and 14 themes through discussion until consensus was reached. The validity of the organization of meaning units, themes, and thematic clusters was verified twice by two external experts. Table 2 shows the three level codes for one of thematic clusters.
6. Validation of Findings with Participants (Member Checking): The thematic clusters and findings were shared with participants to ensure their accuracy and credibility.
7. Supporting Findings with Participant Quotes: Direct quotes from participants were included to support the thematic clusters and allow readers to verify the interpretation and analysis of the data.

All data was generated and analyzed in Korean, then translated into English by a professional qualitative research translation agency. The translated data was verified through back-translation procedures. The translated data was subsequently reviewed for semantic accuracy by a researcher holding a master’s degree from the United States.

### 2.5. Validation and Rigor

To ensure the quality of our qualitative research, we followed Lincoln and Guba (1981) trustworthiness criteria [42]: (1) credibility: the credibility of the research was ensured through peer review and the selection of participants with diverse experiences. The interview transcripts and analysis results were shared with two participants to confirm alignment with their experiences. Researchers independently coded the transcripts, and discrepancies were resolved through discussion and consensus for investigator triangulation. Two versions of the codebook were created to document coding decisions, which served as the audit trail throughout the analysis process; (2) transferability: it was strengthened by maximizing sample diversity, clearly identifying participants, and providing detailed information on the sampling method, as well as the timing and setting of data collection, thus allowing the findings to be applicable in similar contexts; (3) dependability: detailed records were maintained, including interview transcripts, coding processes, and analytic decisions, to ensure transparency. For codes where researchers disagreed, each shared their interpretative basis, and the study’s rigor was enhanced through a consensus meeting. Before explicit analytic decision reporting, two professors with extensive expertise in qualitative research each conducted two rounds of external audits to enhance the study’s quality. The same interview guide was applied consistently across all participants; (4) confirmability: transcription began immediately after each interview. Rich descriptions were also provided to ensure that the research process was clearly understood, which supported the objectivity of the study and the data-driven nature of the findings.

### 2.6. Ethical Considerations

Prior to collecting data, ethical approval was obtained from the Research Ethics Committee of the Korea National University of Transportation (IRB No. KNUT-2025-HR-04-13). To ensure voluntary participation, recruitment was carried out by a researcher unaffiliated with the applicants’ department, thereby maintaining impartiality in the selection of the participants. Immediately before the interviews, the researcher provided a detailed explanation of the study’s objectives, interview procedures, key questions, and expected duration. The participants were asked to provide written informed consent affirming their voluntary involvement. The rationale for audio-recording the interviews and taking field notes was thoroughly explained, and consent for these procedures was obtained. Since focus groups inherently involve sharing information, a clear confidentiality agreement was established to protect participants’ privacy. After recording, only the researchers had access to the materials, and participants were informed that the recordings would be destroyed once the study concluded. In cases where there was potential harm to participants, identifying information was collected with great care. Before the study began, participants were informed that they could withdraw their consent at any time. The researcher recorded reading the consent statement aloud to each participant during their interviews and then referred to each participant by number. Afterwards, it was confirmed that the participants understood and agreed to the consent statement. Additionally, recruitment materials, recordings, and other related records were securely stored in a locked safe within separate offices, apart from the researcher’s office. To alleviate any concerns about potential repercussions related to their student status and encourage candid responses, the interviews were conducted by researchers unaffiliated with the participants’ institutions. Additionally, participants were able to review the recorded materials and transcripts. If any names or other types of identifying information (for example, content specifically mentioning individuals, places, etc.) were present, they could request corrections or deletions. If participants felt discomfort or emotional distress during the discussion, arrangements were prepared—with the participant’s consent—to provide administrative support or access to professional counseling services.

## 3. Results

According to the analysis conducted, 23 meaning units, 14 themes, and seven thematic clusters were identified in relation to nursing students’ experiences of bullying during clinical practice (Supplementary Table S1). The seven thematic clusters include: (1) exposure to harsh speech; (2) experiencing physical harm; (3) being considered a sexual object; (4) disrespected as a nursing student; (5) assigned tasks beyond an individual’s capabilities; (6) restricted educational opportunities during clinical practice; and (7) discriminatory treatment due to healthcare professionals’ prejudice (Table 3).

### 3.1. Thematic Cluster 1: Exposure to Harsh Speech

The first thematic cluster included themes such as “feeling like a target of verbal abuse” and “being belittled with words.” Students in vulnerable positions experienced verbal threats from patients receiving care service or nurses and doctors holding positions of power.

#### 3.1.1. Theme 1.1. Feeling Like a Target of Verbal Abuse

The participants as nursing students reported having unexpected verbal insults or abuse in providing nursing care from patients who were in the position of nursing service recipients. Also, students reported being backbitten by nurses.

*While changing the patient’s clothes, the patient said, “bitch, what are you trying to do by taking off my clothes?” I replied, “Uh, sir, it’s not like that. You need to change into this hospital gown.”*

(G1, P5)

*The nurse knew we were behind her, but she said she really didn’t want the student interns to come and that she hated them. After the head nurse left for the day, she told us to go inside and stay there, so we agreed and went in to work on our assignments. Honestly, everything she said was within earshot, and she was speaking badly about us.*

(G2, P6)

#### 3.1.2. Theme 1.2. Being Belittled with Words

Participants stated experiences of verbal aggression such as being scolded, and being humiliated when they could not answer in the case of being unfamiliar with the names of instruments used during practice and were slow to respond due to unfamiliar hospital layouts, or acting differently from the healthcare professionals’ judgment. The following statements received widespread agreement from the members, as evidenced by nods and interjections.

*The patient pulled out their IV, so it had to be reinserted. The nurse spoke quickly and said, “Please just bring two 24-gauge Jelco catheters,” but since I had never heard that term before, when I repeated, “Jelco?” the nurse responded, “You don’t even know that?” I told her it was my first time hearing the term, and I felt like she was somewhat dismissive of me because I’m a student.*

(G1, P7)

*I went to the fridge to get an ice pack, and the doctor at the station saw me and said that I was just walking back and forth for no reason. I just smiled it off and took the ice pack, but it did make me feel a little uncomfortable.*

(G3, P6)

### 3.2. Thematic Cluster 2: Experiencing Physical Harm

The second thematic cluster included themes such as “uncomfortable touching” and “being physically attacked.” Participants described the experiences of being physical assaulted ranging from touching to physical aggression in clinical settings.

#### 3.2.1. Theme 2.1. Uncomfortable Touching

The participants experienced having a part of their body touched by a nurse in a clinical context and reported feeling embarrassed and disrespected.

*The nurse said something along the lines of, “This is how clinical work is, so make sure you understand,” and kept tapping my hand like this, telling me not to behave that way in a clinical setting. It was my first clinical practice in my third year, and going through that experience made me very reluctant to continue. It also left me with a negative impression of the hospital and completely destroyed my motivation.*

(G2, P7)

#### 3.2.2. Theme 2.2. Being Physically Attacked

Participants often experienced physical threats such as being hit unexpectedly or grasped forcefully by patients when providing nursing care.

*While assisting a patient with changing clothes, I said, “Sir, you need to change into a hospital gown. Could you please move your arm like this?” However, the patient slapped my arm and cursed at me during the process.*

(G1, P5)

*In a situation where I need to get close to the patient, but suddenly (the male patient) grabs my wrist like this. …*

(G2, P1)

### 3.3. Thematic Cluster 3: Being Considered a Sexual Object

The third thematic cluster included themes such as “exposed to unwelcome sexual behavior and inappropriate gazes” and “exposed to unwanted sexual conversations.” The results described situations in which participants encountered sexual objectification such as sexual harassment in clinical settings and felt uncomfortable.

#### 3.3.1. Theme 3.1. Exposed to Unwelcome Sexual Behavior and Inappropriate Gazes

A few students experienced sexual harassment including physical touch and sexual glance with sexual intent from patients while providing nursing care. The students tried to distance themselves from the patients and felt uncomfortable. In this discussion, a few speakers from two groups made statements while the other participants listened silently.

*I was wearing a name tag on the left side of my chest, but the patient kept touching it, possibly out of curiosity about my school or wanting to know my name. Since it was placed on my chest, it felt quite uncomfortable.*

(G1, P3)

*The patient scanned me up and down with his eyes, displaying such behavior, but he neither spoke to me nor acted directly toward me. During the handover, I heard that the patient had made a sexual joke to a nurse, and the behavior directed at me personally was this kind of eye scanning.*

(G2, P1)

#### 3.3.2. Theme 3.2. Exposed to Unwanted Sexual Conversations

A few students also encountered embarrassing situations in which they did not want to respond to sexual questions from a healthcare provider in an isolated case or to a sexual joke from patients. One speaker’s voice nearly broke as she choked up and eventually started crying, and the other participants deeply empathized and comforted the crying participant. Statements about sexual jokes or conversations were mentioned by three students across two groups. Although additional time was provided to encourage more statements, no further comments were made. However, participants were engaged with it naturally, easily forming a sense of empathy.

*Then the doctor asked me, “How old are you?” When I said I was 20 years old, he replied, “Isn’t it better to be with capable men in their 50s rather than young guys around 20?” He insisted that I shouldn’t date men my own age and kept pushing the idea that men in their 40s or 50s are much better.*

(G3, P6)

*Some male patients consistently chose only the nursing students and frequently made sexual jokes. I began to feel uncertain, questioning whether we really need to go this far to satisfy the patients.*

(G2, P1)

### 3.4. Thematic Cluster 4: Disrespected as a Student

The fourth thematic cluster includes themes such as “being treated like an invisible person in an unfamiliar clinical practice setting,” “being treated as a menial laborer without any rights”. The results describe situations in which students feel ignored or treated as auxiliary labor, without recognition of their educational role in the clinical context.

#### 3.4.1. Theme 4.1. Being Treated Like an Invisible Person in an Unfamiliar Clinical Practice Setting

Participants went to clinical practice sites with expectations of clinical practice education and simultaneously felt anxiety about the unfamiliar environment, but experienced indifference, neglect, isolation, and loss of information about clinical practice by the nurse as an educator. When students found that their status as learners was not respected within the clinical nursing settings, participants often experienced distress, withdrawal, lowered self-esteem, and doubts about the nursing profession.

*I entered and said “Hello,” but none of the nurses acknowledged my greeting. I thought I might have spoken too softly, so I tried saying hello again to one of the nurses, but she just gave me a look. … [omitted] … No one informed me where I was supposed to go, and there wasn’t even a nurse in charge.*

(G1, P5)

*When we wanted to learn something and followed a nurse, she was very unfriendly and seemed irritated, as if we were just a nuisance, which made it quite difficult for us.*

(G2, P6)

#### 3.4.2. Theme 4.2. Being Treated as a Menial Laborer Without Any Rights

The participants stated that, during clinical practice, when their names were called, they were addressed informally without respect by nurses or patients, and caregivers. Additionally, basic student rights that should be ensured during clinical practice—such as designated mealtimes, break time, and working hours, including the start and end times of practice—were not upheld. Students had an orientation about institution for practice and working hours including meals time, break time, the start and end times of practice, and calling others, etc., in school before clinical practice, but students worked overtime and did not have the minimum rights as a student.

*It appears that the nurse uses titles very informally, which comes across as quite disrespectful. At the very least, nursing students should be treated with respect, but the way these titles are used doesn’t convey that at all.*

(G3, P7)

*But even when our shift officially ends at 9 o’clock, and we’ve finished everything by 8:55, suddenly, around 8:59, the nurse assigns us one more task and says, “Do this now.” As students receiving these last-minute tasks, we honestly can’t refuse.*

(G2, P2)

### 3.5. Thematic Cluster 5: Assigned Tasks Beyond an Individual’s Capabilities

The fifth thematic cluster includes themes such as “facing a dilemma of choice due to conflicting instructions given simultaneously” and “being forced to perform tasks that are difficult to handle.” The results describe situations in which students are forced to perform assigned tasks due to hierarchy, despite lacking the knowledge and experience compared to nurses.

#### 3.5.1. Theme 5.1. Facing a Dilemma of Choice Due to Conflicting Instructions Given Simultaneously

The participants performed nursing tasks under the direction of one healthcare professional while simultaneously receiving instructions from another. In attempting to resolve such conflicts, they are in a position of weakness faced with the difficult attitude of a dominant figure who, without considering the student’s position, insisted that their instruction take priority. Moreover, they were uncertain about how to proceed and to have choice. These situations within a rigid hierarchical structure could make students experience psychological distress, lowered self-esteem, and doubts about the nursing profession.

*The nurses asked me to complete a task, so I was working on it. Then, another nurse requested something different. Since my hands were full, I was about to set the tray down and leave, but the second nurse said, “Student, why aren’t you leaving? You’re not supposed to be here.”*

(G1, P6)

*The caregiver asked if I could check it briefly. I stayed there for only a short time, but the head nurse saw me in my caregiver’s ward. Then, the head nurse said, “Do you not think what I said makes sense? I told you to watch it, so why did you not?” I explained what had happened, but she dismissed it. She added, “I’m the head nurse, and you’re a student, so shouldn’t you prioritize my instructions?”*

(G3, P7)

#### 3.5.2. Theme 5.2. Being Forced to Perform Tasks That Are Difficult to Handle

Participants stated experiences of being assigning tasks that were not within student competence in the hierarchical structure.

*The nurse sent me to a patient who wasn’t following her instructions. She said, “This patient is hard to communicate with. You handle the patient interview and schedule the outpatient appointment. Do your best.” It was really tough.*

(G1, P1)

### 3.6. Thematic Cluster 6: Restricted Educational Opportunities Corresponding to Clinical Practice Objectives

The fourth thematic cluster includes meaning units related to obstacles encountered during practical training such as “conformity to avoid evaluation-related disadvantages” and “restricted clinical practice due to a lack of trust in students.” These results describe situations in compliance with clinical practice that do not correspond to the purpose of practical training due to concerns regarding an evaluation of their grades and school from the nurse as an evaluator.

#### 3.6.1. Theme 6.1. Conformity to Avoid Evaluation-Related Disadvantages

Students placed a high priority on achieving good grades and had a strong desire to impress the nurse evaluator with power and have positive evaluations during clinical practice. Therefore, even when nurses assigned tasks beyond their scope, students were reluctant to refuse them and instead accepted the extra work. Furthermore, they performed unwanted tasks out of concern for maintaining their schools’ internship program and avoiding negative consequences for other students. Participants reported internal difficulties such as emotional depression, lowered self-esteem, and skepticism toward the nursing profession due to concerns about grades, school reputation, and reduced opportunities.

*Initially, we were only required to do it once, but she (nurse) insisted on another go. Consequently, I had to prepare all over again, and since I was managing other tasks as well, it was quite exhausting. This requirement was unique to my ward—it wasn’t enforced elsewhere—so I felt it was unfair. Since we were the ones being evaluated, we couldn’t refuse and had to comply, but it still felt somewhat unjust.*

(G2, P1)

*I worry that if I make a mistake, it could negatively impact the students who come after me, and if the professor finds out, it might even affect my grades. As a result, I tend to endure it. The professor probably wouldn’t say it outright, but I might hear comments like, “Because of you, the institution for clinical practice is no longer available for training.” The professor also shares stories about students who lost clinical practice opportunities due to their behavior. It feels like the underlying message is, “Just do a decent job and don’t cause any trouble”.*

(G3, P5)

#### 3.6.2. Theme 6.2. Restricted Clinical Practice Due to a Lack of Trust in Students

The participants reported that patients and caregivers do not trust students who have not yet obtained nursing licenses, leading to refusal of care, even for simple nursing tasks such as taking vital signs. Additionally, in clinical environments—where it is already difficult to practice nursing tasks directly—students’ opportunities for indirect observation were limited because nurses were reluctant to allow students to observe their nursing practices.

*When I take the vital signs of patients, they often don’t trust me because I’m a student. They say things like, “Call a nurse. Why are you doing this? Aren’t you just a student?”*

(G2, P6)

*The nurse told me not to come near during nursing care or procedures because it’s disruptive. She seemed annoyed and asked if anyone had informed me about this before.*

(G1, P2)

### 3.7. Thematic Cluster 7: Discriminatory Treatment Due to Healthcare Professionals’ Prejudice

The fifth thematic cluster includes themes such as “being treated unequally when the training hospital is not affiliated with the students’ school” and “being asked to practices based on gender stereotypes.” Students reported experiencing discrimination in educational opportunities depending on their academic affiliation and gender.

#### 3.7.1. Theme 7.1. Being Treated Unequally When the Training Hospital Is Not Affiliated with the Students’ School

The participants reported that, during their clinical training at hospitals other than their affiliated hospital, they encountered fewer educational opportunities for practical training compared to students training at their affiliated hospital. Additionally, they experienced differential treatment from healthcare providers based on their school affiliation. Participants described that medical personnel treated students differently, resulting in discrimination in educational opportunities according to the academic background of the medical personnel.

*The nurse seemed to understand and told me, “I’ll schedule you for an earlier training session,” then left. I ended up waiting for over 30 min, but in the end, I couldn’t attend the training at all, and they didn’t offer me another opportunity. When I reflected on why they called me instead of other students from my unit, I realized they assumed I didn’t need to attend because I’m not one of their alumni.*

(G2, P5)

*She (nurse) explained that she had a very close connection with the students because they all graduated from the same school as she did. This close relationship seemed to imply that they were from the same school. Because of that, they received very favorable treatment; even if they asked an incorrect question, the nurse would say something like, “I’ll speak with the professor so you can understand it better.” However, if we could not answer, she would say, “Doesn’t your school teach you this?”*

(G3, P7)

#### 3.7.2. Theme 7.2. Being Asked to Practices Based on Gender Stereotypes

Each of the male students reported being selectively assigned physically demanding duties and having more presentation opportunities than female students. When a statement was made that assigning tasks requiring physical strength to male students is unfair, it was observed that the majority of the female students in the group agreed. Moreover, clinical practice for male students was sometimes limited compared to female students when providing care to female patients. Although students desired equal clinical practice education, they experienced different opportunities in some aspects of clinical practice based on gender and perceived this as discrimination. However, data collection was limited due to the small number of male student participants.

*I’m a male student, as you know. During one of my clinical rotations, the transport staff needed to move a patient. They asked me to wheel the patient to the testing room and then return. I understand their reasoning, but it might seem somewhat discriminatory. Since most nurses are women, I think male students face some disadvantages. On the other hand, when tasks require physical strength, male students are usually called upon first.*

(G3, P2)

*Male students were often prohibited from watching procedures such as Foley catheter insertions on female patients. However, when male patients received Foley catheters, all students observed together.*

(G3, P5)

## 4. Discussion

We examined experiences of bullying using seven thematic clusters. A discussion of the research findings is organized around each of these clusters.

The first thematic cluster, “Exposure to harsh speech”, revealed that participants reported experiencing verbal abuse from individuals in the hospitals during their clinical training. Participants encountered abuse not only from patients and their caregivers but also from nurses and physicians. In South Korea, among workers in the medical, educational, and financial service sectors, medical workers experience harassment at a rate approximately two to three times higher than that of other occupational groups [24]. And the prevalence of verbal violence experienced by nursing students during clinical practice in South Korea ranges from 73.3% to 98.3%; the perpetrators of verbal abuse were primarily patients. Among the types of bullying committed by hospital staff, including nurses and doctors, verbal abuse was the most common [22]. Students are exposed to increasing levels of bullying as their clinical practice duration lengthens and their grade level advances. Consequently, their awareness of violence grows, resulting in stronger emotional responses [22]. The attributes of bullying categorized as emotional abuse—such as intimidation, threats, and insults—have the most severe impact among all types of bullying [5]. Four qualitative studies conducted in South Korea commonly report the severity of students’ experiences of verbal abuse during clinical practice [22]. Bullying leads to numerous negative physical, mental, and social consequences [3,12,13,14]. However, recent studies in Korea report that verbal abuse continue to persist [14,20,21,22,29,30,31,33]. The failure to eliminate verbal abuse directed at nursing students in clinical settings can be attributed to inadequate measures and insufficient policies. Although 92.9% of the students expressed a need for education on how to prevent verbal violence, only 23.6% of educational institutions and 14% of clinical practice sites offer training on how to cope with violence [22]. Early recognition of bullying and appropriate intervention strategies effectively reduce its occurrence [43]. Previous research on service occupations, including hospitals in South Korea, found that 66.4% of workplaces were ineffective in addressing harassment. Among these, 21.5% had no coping strategies in place. Notably, there was a lack of comprehensive and proactive institutional improvements, such as the development of policies related to coping, the establishment of clear measures against perpetrators, and the implementation of incident reporting systems [24,25]. These are environments where bullying continues tend to normalize its presence [24]. It is essential to find ways to disrupt this normalization. Consistent with these results, nurse managers and educators have emphasized early intervention in preventing the normalization of bullying [11]. Consequently, it is imperative to implement educational programs that target nursing students at the start of their academic training to prevent bullying and related victimization.

The second thematic cluster is “Experiencing physical harm”. Participants in this study reported being subjected to attacks such as having their wrists grabbed or being hit. According to the literature, physical violence is the least frequently experienced type of violence among nursing students during clinical practice in South Korea, with reported rates ranging from 18.6% to 25.8%. Only a small number of participants in each group reported such experiences, consistent with previous research. Emotional responses tend to intensify after experiencing violence [22], stress levels increase [44], and it negatively affects the professional image of nursing, career decisions [28], and career attitude maturity [33]. Although only a few participants were exposed to physical violence, those who reported it expressed feelings of embarrassment due to the threatening physical contact and confusion about how to respond. The perpetrators were mainly patients and nurses. Physical violence can cause physical harm, and although its frequency is low, its severity can be high depending on the situation. However, students’ most common coping strategy toward violence was “not responding to the perpetrator and continuing clinical practice” [22,33]. Harassment is often underreported and thus tends to be a “silent epidemic” [45]. Yoo identified several reasons for underreporting, including victims’ concerns that reporting group bullying might worsen the situation or that no action would be taken, worries about confidentiality, fear of becoming a scapegoat, concerns about being labeled a troublemaker at work, fears of being perceived as accepting failure, and learned tolerance that normalizes bullying behavior [24]. Additionally, victims tend to underreport harassment due to fear of retaliation from employers, colleagues of the perpetrator, or influential professionals; those in higher positions can jeopardize the careers of whistleblowers [46]. In particular, clinical practice instructors who are nurses hold relatively high positions and exert influence over students’ learning; thus, when the perpetrator is a nurse, students face greater difficulties in coping. Therefore, in clinical settings, strengthening sanctions against perpetrators through a zero-tolerance policy can reduce the normalization of harassment. Moreover, implementing a reporting system that guarantees victims’ confidentiality can help reduce underreporting.

The third thematic cluster is “Being considered a sexual object”. A literature review reported that 14.2% to 47.5% of nursing students experienced sexual bullying during their clinical practice. While verbal abuse and physical threats were found to be more prevalent during students’ clinical training, sexual violence followed in frequency [22]. Among the study participants, few were male, and only a few female students reported sexual harassment. However, previous research indicates that male nurses have also experienced sexual harassment [22,47]. Thus, sexual objectification should be recognized as a gender-transcending issue. Despite stricter social regulations, sexual harassment continues to be reported in South Korea, largely. Hospitals prioritize job dissatisfaction and high turnover rates among nursing staff. These aspects of the South Korean medical environment present significant challenges for nurses. Experiences of sexual harassment are highly sensitive and often go unreported due to societal reluctance to disclose such issues in South Korea. Although studies indicate that sexual violence is more common among nurses than nursing students in clinical settings, the importance of maintaining a safe practice environment for students has been emphasized [22]. In this study, since cases were shared among members through focus group interviews, it is possible that participants experienced sensitive issues such as sexual bullying but chose not to disclose them. All participants had completed mandatory annual sexual violence prevention education at their respective schools, which heightened their gender sensitivity. In the Korean higher education system, students receive significant protection at home and school, but clinical settings offer fewer protective measures, which seems to amplify the impact of negative experiences. Although many participants did not explicitly state it, considering the potential severity and risks of sexual bullying experienced by students in clinical settings, in addition to implementing a reporting system, policies must be established for all individuals within the hospital environment, including patients, medical staff, and nursing students. There should be open social discussion and consensus regarding re-education about sexual abuse, as well as the development and operation of counseling programs for both victims and perpetrators.

The fourth thematic cluster pertains to “disrespected as a student”. The participants encountered situations where they had to endure nurses’ disregard and rudeness, feeling burdensome like luggage. Although the students had names, they were often addressed without them, and meal times or clock-in/out times were frequently ignored, resulting in overtime work. The students reported being treated as if they did not exist. Basic regulations such as practice hours, meal times, and rest periods were frequently not respected. These findings were derived from a systematic review of the qualitative literature and partially align with previous studies [14,23,27,29,30,31,33]. Prior research similarly reported students feeling ignored and being called inappropriate titles instead of their names [29]. Poor resolution of rudeness, which is sometimes conflated with bullying, can lead to workplace bullying and eventually physical abuse [48]. Even nurses often become targets in hostile environments; although they may be able to cope, they become victims of bullying behaviors due to power imbalances and a lack of coping skills or refusal [43,48]. In South Korea, large general hospitals preferred by nursing colleges often handle severely ill patients, which can exacerbate emotional tension between patients and their families. Although many medical staff are present, the heavy workload makes detailed guidance difficult [22]. Nursing staff have both positive (enabling) and negative (hindering) effects on students’ clinical learning and socialization into nursing. Nursing staff may encourage and motivate students when acting as positive mentors and facilitators. However, their actions can also negatively affect students by decreasing their confidence, learning, and desire to continue in the profession [49]. Clinical practice institutions and nursing colleges must recognize the seriousness of bullying experienced by nursing students. Experiences of being ignored and receiving no guidance in a tense medical environment cause students to feel depressed and anxious [27]. Bullying has psychological and economic impacts, and some students even consider quitting nursing due to these harmful effects [14,20]. These impacts are not limited to the learning period but can lead to long-term consequences such as embarrassment, fear of further repercussions, and deterioration in the quality of future patient care [22]. A literature review addressing bullying between nurses and clinical nursing leaders proposed response strategies such as raising nursing students’ awareness of their rights, improving their ability to recognize appropriate feedback channels when bullied, and strengthening nursing leadership supervision systems [14]. Since organizational factors have a greater influence on bullying than personal factors [11], clinical practice sites should conduct investigations and implement measures to foster a positive organizational nursing culture. Additionally, nursing colleges should provide various extracurricular programs to help students develop personal coping strategies to manage psychological difficulties and facilitate recovery.

The fifth thematic cluster pertains to “assigned tasks beyond an individual’s capabilities,” suggesting that these concerns are grounded in reality. This finding partially corroborates earlier studies that documented students being assigned tasks beyond their skill level or being overwhelmed by excessive workloads [13,29,50]. Harassment occurred within a rigid hierarchical organizational culture, with nursing students expressing feelings of powerlessness in clinical environments dominated by nursing staff, often describing a sense of resigned acceptance [50,51,52]. Participants reported instances such as the simultaneous assignment of multiple tasks and the enforcement of work priorities within a hierarchical structure. Students experienced the limitations of their personal abilities due to their social position as students. They expressed conflicts and stress in these situations. These findings were derived from a systematic review of the qualitative literature and partially align with previous studies [14,23,27,29,30,31,33]. Past research also identified stress caused by unrealistic deadlines [19]. A systematic review classifying excessive work demands on students [14] revealed similar patterns, including the requirement to perform multiple tasks and the expectation to follow nurses’ instructions within hierarchical relationships. Previous studies conducted in Korea reported that nursing students perceived themselves as occupying “the lowest position among hospital staff” and highlighted role identity confusion stemming from vertical power dynamics within the organization, where “the hospital is dominant and the school is subordinate,” forcing them to endure unfair treatment [20,22]. Participants in this study clearly expressed sentiments such as, “If we are treated like this while paying clinical practice fees, wouldn’t it be better to practice at school?” This appears to be a cynical expression of the negative emotions and stress students experience within the rigid hierarchical order of hospitals. The reason nurses assign overwhelming tasks to students during clinical practice without providing leeway can be inferred from previous studies. The bullying phenomenon known as “taeum,” commonly observed in Korean nursing settings, mainly occurs when senior nurses train new nurses. Causes include the absence of formal training programs, nurse shortages, and the perception of “taeum” as a rite of passage for new nurses [3]. Even nurses with professional expertise report physical and mental stress, including turnover, due to bullying [3,11,12,13,24,51]. Sometimes, bullying occurs without any apparent reason [11]. The nursing field, which regards such unfair bullying as a rite of passage that new employees must endure, can have direct and negative effects on nursing students. Considering that nursing students are future new nurses and that new nurses eventually become senior nurses, organizational efforts within the nursing field are necessary to break this vicious cycle of bullying. Nurses in clinical settings may face challenges comparable to those of nursing students due to nurse shortages and the need to share work with inadequately trained students. Therefore, nursing organizations should consider implementing standardized educational guidelines in clinical settings that align with the educational objectives for nursing students. Since individuals perceive their organizational culture as more relationship-oriented, they experience less bullying. Therefore, it is necessary to cultivate a relationship-oriented organizational culture grounded in trust, justice, and fairness [11]. Additionally, promoting small-scale clubs and cultural events with diverse organizational purposes can help foster relationship-oriented human connections.

The sixth thematic cluster is “restricted educational opportunities corresponding to clinical practice objectives.” Hostile behaviors such as restrictions on opportunities for practice or the refusal to assign nursing tasks (as reported by the participants) are consistent with the findings of past research [15,31,53]. Students reported that, due to concerns about practical evaluations, they felt compelled to comply with unexpected instructions, even when they perceived them as unfair, and were unable to refuse such directives. This aligns with prior research indicating that students sometimes receive poor grades as a punitive measure [13]. Although participants in the present study said they reluctantly accept assigned tasks, some do so out of concern that negative evaluations of clinical sites by their affiliated schools might harm the institution’s reputation or jeopardize future clinical placements, an aspect not previously documented in previous studies including systematic reviews on bullying [17,19,22]. Contrary to the findings of a prior study [47] in which students expressed satisfaction by assertively refusing to perform tasks, no participants in this study reported such refusals. These findings underscore the need for enhanced assertiveness training. Nurses and nursing leaders may respond negatively when tasked with educating students amid excessive workloads and staffing shortages. It is essential to prioritize the provision of clear, specific guidelines for clinical practitioners and faculty members regarding the scope and content of clinical practice prior to students receiving training. While requiring careful deliberation, implementing measures that hold nurses and nursing leaders accountable for bullying individuals in subordinate positions within educational and clinical training settings may also be warranted [14,26]. It may be possible to explore ways to benefit nurses who supervise students’ clinical practice in terms of their career development or to enhance the quality of education within the nursing organization by having students evaluate their satisfaction with the clinical practice institution and provide feedback to hospital staff.

The seventh thematic cluster pertains to “discriminatory treatment due to healthcare professionals’ prejudice.” The participants reported instances of discrimination during clinical practice, particularly when the training hospital was not affiliated with their school. Such discrimination manifested as a differential treatment by nursing staff based on students’ institutional affiliation. While previous research has documented racial discrimination in clinical settings [54,55], the present findings reveal additional dimensions that have not been addressed previously. The literature exploring the antecedents of bullying suggests that students lacking affiliation with training hospitals report higher rates of violence; contributing factors include nursing staff shortages, excessive workload, organizational tolerance to bullying, and suboptimal nurse quality [3,11,14]. In South Korea, the rapid expansion of nursing programs has resulted in a shortage of clinical training sites, creating a power imbalance between educational institutions and clinical training facilities. Furthermore, South Korea’s sociocultural emphasis on alma mater prestige in social interactions has been posited as influencing these outcomes. We also found that male nursing students have experienced restrictions on clinical practice and have been assigned physically demanding tasks based solely on their gender, corroborating findings from recent South Korean research [56]. However, such occurrences remain relatively uncommon. Nursing in South Korea is traditionally regarded as a female-dominated profession; by 2025, the number of female nurses is 491,174, whereas the number of registered male nurses is 35,764, constituting approximately 7% of all nurses [57]. Although female nursing students have been identified as more susceptible to violence, male students reported experiences of patient and caregiver rejection as well as challenges related to gender identity, which they described as burdens borne individually [22,23]. The rising enrollment of male nursing students has heightened the need for effective management of clinical practice-related stress and underscores the need for expanded research focused on this demographic [22,23]. Despite this, the paucity of quantitative and qualitative studies addressing male nursing students’ clinical experiences indicates a pressing need for further investigation. Bullying and discriminatory experiences reported by nursing students during their clinical placements are likely to adversely affect their professional trajectories. Consequently, it is critical to develop a discrimination prevention training program for clinical practice instructors.

In South Korea, research on bullying among nursing students remains very limited. Particularly in Southeast Asian countries with strong hierarchical cultures and power dynamics in clinical settings, reporting bullying is often suppressed, making it difficult to accurately measure its incidence and impact [58]. Unlike Western cultures that emphasize individual rights, South Korea has a Confucian tradition that prioritizes social status and age. The prevailing social atmosphere, which regards patient satisfaction as the hospital’s top priority and assumes the dedication and patience of nurses—who are viewed as service workers—also influences bullying in nursing workplaces. Practical nurses, who play a central role in clinical practice, face numerous challenges but occupy relatively lower ranks in the hierarchy, limiting their ability to lead organizational problem-solving. Nursing students, who rely on the guidance of practical nurses, are socially influenced by them, and experiences of bullying from these nurses may have negative effects, such as decreased self-confidence, reduced desire to pursue nursing professionally, and diminished enthusiasm for learning [49]. This issue is compounded by the vulnerability of students due to the significant power gap between students and clinical staff [59]. Participants have reported that harsh language and behavior from medical professionals and patients toward nursing students, as well as a lack of respect for their roles or status, cause psychological distress and stress, leading to experiences of depression, low self-confidence, and reduced self-esteem. As previous studies have shown, such power imbalances leave students feeling powerless to respond to bullying, often leading them to remain silent to avoid conflict or to maintain their position within the clinical environment [14,20,22]. Moreover, sexual harassment, which is taboo in Eastern cultures, causes serious secondary damage as victims are socially stigmatized, further silencing nursing students and complicating accurate assessment of the problem. Therefore, additional research on sexual harassment during clinical practice among nursing students is urgently needed. Identity confusion among nursing students is also a major concern; statements such as “Because the hospital is the superior and the school is the subordinate, one must endure unfair treatment” may underlie outcomes such as discrimination based on students’ school affiliation and gender, or enduring clinical practice experiences that do not align with educational objectives. Previous studies have noted that fear of retaliation or disadvantages from those in power causes hesitation in reporting or confronting bullying behaviors [14,24]. This not only affects students’ wellbeing but also negatively impacts the quality of patient care they provide [20,22]. This phenomenon illustrates the profound impact bullying has on nursing practice. Therefore, changes in nursing education policy are necessary, along with the development of specific policies and comprehensive support systems for nursing students.

### Limitations

This study has several relevant limitations. For instance, the use of focus groups may have introduced social desirability bias and inhibited participants from sharing more sensitive experiences. Given the sensitive nature of the topic, data collection through focus groups might have led to self-censorship. Additionally, interactions within the group could result in dominant speakers influencing others, while the silence of passive participants might have undermined the diversity of the data collected. Additionally, snowball sampling could have introduced bias during data collection. The small number of male participants, the inclusion of only two educational institutions, and the specific cultural context of South Korea increase the probability of underreporting in the research results. Additionally, considering the nature of the topic and the group interview setting, there is a possibility of underreporting sensitive issues such as sexual harassment. The manuscript also has the limitation of making broad conclusions based on data from only two institutions and three focus groups, the limited member checking and the conceptual overreach in labeling diverse harms as bullying. Additionally, the confidentiality agreements implemented within the focus groups to protect participants’ sensitive information limited the disclosure of the collected data. Lastly, the interchangeable use of terms such as bullying, harassment, and violence without clear definitions represents a limitation of this study.

## 5. Conclusions

This study provides an in-depth understanding of bullying by describing South Korean nursing students’ experiences such as exposure to harsh speech, experiencing physical harm, sexual objectification, restricted educational opportunities, and discrimination during clinical practice. The results of this study not only provide data for understanding the bullying experienced by three focus groups of South Korean nursing students within the cultural, institutional, and educational contexts of South Korea during clinical practice, but also provide a clearer reflection on implications for educational policy or clinical practice.

These findings highlight the importance of recognizing bullying experiences during clinical practice. Such a recognition is essential to support students in effectively managing bullying and to promote the development of a positive professional nursing identity. Based on the results of this study, several recommendations are proposed. First, universities should acknowledge the severity of bullying that nursing students may encounter during clinical practice and develop comprehensive programs to help students cope with these challenges. Second, clinical practice institutions should actively foster respect for human rights through bullying awareness training programs, intuitional reporting system, or structured supervision strategies during clinical practice. Finally, further research on bullying experienced by Korean nursing students should be conducted repeatedly. Although this study reflects recent experiences, ongoing in-depth research is needed to improving the quality of nursing education in Korea and effectively addressing bullying during clinical practice.

## Figures and Tables

**Table 1:** Interview guide.

Starting question	What ideas come to mind when you think of bullying?
Main question	What is the reality of the bullying you experienced during clinical practice? - Probe: What memories come to mind regarding the bullying of vulnerable individuals that you experienced during clinical practice? - Probe: How did you feel when you were bullied? - Probe: What was your coping response when you were bullied? - Probe: How has this experience affected your approach to clinical practice?
Conversion question	Is there any strategy to prevent bullying during clinical practice?
Summary question	Is there any other feedback you can give us related to this topic?

**Table 2:** Examples of 3 levels of coding.

Level 3 Thematic Clusters	Level 2 Theme	Level 1 Meaning Unit
Exposure to harsh speech	* Feeling like a target of verbal abuse	Being cursed by patients while providing care
		Hearing unfavorable assessments of fellow students directly from a nurse nearby
	* Being belittled with words	Being criticized for nursing practices

**Table 3:** Thematic clusters and themes of bullying experiences of nursing students during clinical practice.

Thematic Clusters (7)	Themes (14)
Exposure to harsh speech	Feeling like a target of verbal abuse
	Being belittled with words
Experiencing physical harm	Uncomfortable touching
	Being physically attacked
Being considered a sexual object	Exposed to unwelcome sexual behavior and inappropriate gazes
	Exposed to unwanted sexual conversations
Disrespected as a nursing student	Being treated like an invisible person in an unfamiliar clinical practice setting
	Treated as a menial laborer without any rights
Assigned tasks beyond an individual’s capabilities	Facing a dilemma of choice due to conflicting instructions given simultaneously
	Being forced to perform tasks that are difficult to handle
Restricted educational opportunities corresponding to clinical practice objectives	Conformity to avoid evaluation-related disadvantages
	Restricted clinical practice due to a lack of trust in students
Discriminatory treatment due to healthcare professionals’ prejudice	Being treated unequally when the training hospital is not affiliated with the student’s school
	Being asked to practices based on gender stereotypes

## Data Availability

The data generated and analyzed during this study are not readily available because ethical approval for use relates only to the research team. Requests to access the datasets should be directed to the corresponding author upon reasonable request, subject to ethical approval.

## Supplementary Materials

The following supporting information can be downloaded at: https://www.mdpi.com/article/10.3390/healthcare14091182/s1, Table S1: Bullying Experience Theme Summary.
