# Supplementary material for: Bullying Experiences of South Korean Nursing Students During Clinical Practice: A Focus Group Study on Two Colleges
Source: Healthcare (Basel). 2026 Apr 28;14(9):1182. doi: 10.3390/healthcare14091182 (PMC13163464; doi:10.3390/healthcare14091182)
Supplement: Supplementary file 1 [file healthcare-14-01182-s001.zip › healthcare-4186877-supplementary.pdf]

Table S1. Bullying Experience Theme Summary.

Theme Clusters (7)	Themes (14)	Meaning Units (23)	Statement (Examplars)
Exposure to harsh speech	Feeling like a target of verbal abuse	• Being cursed by patients while providing care	The patient cursed at me, saying, "What the hell does it have to do with you?" (G1, P5)<br>A patient was wearing sunglasses. The head nurse with me asked the patient why he was wearing them in the wards, and the patient responded with, "What the hell, bitches!". The patient then continued to curse repeatedly, saying "shit, shit, shit" (G1, P5)
		• Hearing unfavorable assessments of fellow students directly from a nurse nearby	The nurse says things like, "The students who came last time weren't very good," where we can hear her. (G1, P1)<br>After the handover ended, I was standing with other students before going on rounds behind the nurses. One nurse, aware that we were standing behind her, said, "I really hate nursing students. I wish they wouldn't come." Another nurse replied, "Still, they do take the vitals." Honestly, it was close enough for us to hear everything. (G2, P7)
	Being belittled with words	• Being criticized for nursing practices	I went to the fridge to get an ice pack, and the doctor at the station saw me and said that I was just walking back and forth for no reason. I just smiled it off and took the ice pack, but it did make me feel a little uncomfortable. (G3, P6)<br>At that time, the nursing process involved reviewing the patient chart and calling out each patient's name. There were so many people that I couldn't hear anything clearly. If the work was even slightly delayed, the nurse would scold me, saying, "Why can't you even call out the names? Shouldn't you be calling them ahead of time, quickly, quickly?" (G3, P5)
Experiencing physical harm	Uncomfortable touching	• Being tapped on the hand by a healthcare worker while providing an explanation	When the nurse first said that clinical practice and theory are different, I thought she said it because I didn't know much. I also thought she might have been a bit annoyed having to explain things to me since nurses are busy. But when she slapped her fingers like that, I felt bad and truly felt disrespected. (G2, P7)<br>I can tell whether the feeling is good or bad. The nurse was tapping my hand and talking to me to test how I would react. (G2, P1)
	Being physically attacked	• Being hit by a patient while providing care	When the patient hit my hand and cursed while I was helping them change clothes, I thought, "If it's going to be like this, and I'm paying for the practice, wouldn't it be better to do the practice at school?" (G2, P7)<br>I went to a ward with many elderly patients, and the nurses knew that I was getting hit by the patients. (G2, P5)
		Being physically overpowered by the patient	In a situation where I need to get close to the patient, but suddenly (the male patient) grabs my wrist like this. ….. (G2, P1)
Being considered a sexual object	Exposed to unwelcome sexual behavior and inappropriate gazes.	• Being subjected to physical contact during nursing tasks	When I need to perform a blood sugar test (BST) or measure blood pressure, I have to get close to the patient. However, the patient suddenly grasped my wrist. (G2, P1)<br>The patients kept touching me. I found it understandable when they touched my arm or back, (omitted) It happened repeatedly. (G1, P3)
		• Being scanned up and down by patients while providing care	The patient would scan me up and down with his eyes. (G2, P1)
	Exposed to unwanted sexual conversations	• Being embarrassed to answer sexual questions	Some male patients consistently chose only the nursing students and frequently made sexual jokes. I began to feel uncertain, questioning whether we really need to go this far to satisfy the patients. (G2, P1)<br>The man (The patient), who was in his 40s, used to make sexual jokes. (G2, P1)
Disrespected as a nursing student	Being treated like an invisible person in an unfamiliar clinical practice setting	• Being ignored by nurses	Well, I greeted them, but the nurses didn't even respond, so it didn't feel welcoming. I thought this must be a hospital with a poor atmosphere. I decided that I simply wouldn't apply here later. (G1, P5)<br>I said hello, but none of the nurses responded to me, so I didn't know what to do on my own. When I opened the shoe rack, I saw many slippers and wondered if it was okay to put mine there. Finally, a nurse asked me what I was doing. I

			told her it was my first time here and that I didn't know where to put my slippers. The nurse told me to just put my slippers anywhere. (G1, P3)
		• Being perceived as a nuisance by nurses	I didn't know exactly whom to ask when I didn't understand something, so I just ended up standing there like a folding screen. I wasn't sure which nurse I was supposed to ask about what, and since everyone seemed busy, I felt like I shouldn't bother any of the nurses. (G2, P6)<br>When I first heard the nurse say not to follow me, I was a bit taken aback. I wondered if the nurse was embarrassed or felt uncomfortable because of me. (G1, P7)
	Treated as a menial laborer without any rights	• Being addressed disrespectfully	When the nurses called me, they used condescending terms such as "hey you" or "you there," as if addressing a small child. (G3, P1)<br>In one ward, a nurse usually addresses me as "student" or but if I don't act quickly, she suddenly changes how she addresses me and says something like, "Hey, aren't you calling the next person?" I felt like she saw me merely as a part-time worker. (G3, P1)
		• Practice hours that exceed the designated limits	Now, when it's time to eat or leave work, the nurses are supposed to send me out on time. However, even when the time came, they didn't allow me leave. As a result, I sometimes ended up staying about 20 minutes overtime. During lunch, when I asked, "Can I go eat now?" the nurses said that they themselves hadn't been able to eat either. (G2, P6)<br>After lunch, I planned to rest briefly in the locker room, but the nurses would occasionally come in and glance at me. Their looks seemed to say, "Are you going to stay here the whole time?" It felt like a form of unspoken pressure. Because of this, I often ended up finishing my lunch break early and wasn't truly guaranteed the full break time. (G3, P7)
Assigned tasks beyond an individual's capabilities	Facing a dilemma of choice due to conflicting instructions given simultaneously	• Receiving nursing task instructions simultaneously from different staff members	The caregiver asked me to help move the patient. After struggling together to finally get the patient seated, I hesitated about whether to continue assisting and ultimately declined to help further. This was because I knew the ward nurse was waiting to assign me tasks and was wondering why I was taking so long. (G1, P6)<br>Students are supposed to leave their lab coats in the places that nurses allow, but since no one said anything, I was holding mine in my hands. One nurse told me to keep it inside, but another nurse said to leave it outside. Not knowing what to do, I once placed it on top of the shoe rack. (G1, P3)
	Being forced to perform tasks that are difficult to handle	• Being compelled to follow instructions within a hierarchical relationship	The nurse sent me to a patient who wasn't following her instructions. She said, "This patient is hard to communicate with. You handle the patient interview and schedule the outpatient appointment. Do your best." It was really tough. (G1, P1)<br>The nurse turned on the light in the empty room and pointed to the desk and chair in the corner, instructing me to "study." I spent about four hours there alone, reading the practice manual. After lunch, she suddenly told me to come out because there was a medical school class. I moved the desks outside again and had to sit and study for three days. (G1, P3)
Restricted educa-tional opportunities corresponding to clinical practice ob-jectives	Conformity to avoid evaluation-related disadvantages	• Accepting unexpected instructions due to concerns about clinical practice evaluations	The nurse gave me an assignment and said something like, "The assignment isn't mandatory, but these things all reflect your attitude scale." It was tough because, in addition to the assignments I had to do for school, I also had to do this. (G3, P2)<br>The nurse asked me to create a report. but I am not sure where it will be used. Because I worry that speaking my mind freely might affect my grades, I end up holding back more and feeling intimidated. (G3, P3)
		• Performing unwanted tasks due to worries about the evaluations of the affiliated school during clinical practice	My school does not have an affiliated hospital, so sometimes hospitals refuse to accept nursing students for clinical practice because previous students exhibited poor attitudes. That is why I have to endure this situation. (G3, P2)<br>Because of some of my behaviors, the next students could also be affected, so I think that's why I just endure it. It's because I might hear things like, "I lost my clinical practice hospital because of you!". (G3, P5)

	Restricted clinical practice due to a lack of trust in students	• Patients and caregivers refusing to receive care	The patient was uncooperative when I attempted to take their vital signs. The patient said, "should I cooperate with you? Just call the nurse" (G1, P1)
			During the BST test, the family members of the elderly female patient surrounded me, all together, repeatedly saying things like, "Why is a student performing this procedure? Is the student even a doctor? How many times are you going to poke her in one day?" I was so flustered that I couldn't respond. (G1, P5)
		• Healthcare providers restricting students' clinical practice opportunities	From a distance, I couldn't clearly see what kind of treatment or nursing care was being provided, so I approached the nurse to take a closer look. However, the nurse said irritably, "Don't come over while I am performing nursing treatment! You're being a distraction! Didn't anyone tell you that?" I unintentionally lost the desire to learn and felt somewhat intimidated. (G1, P2)
			I was listening to a patient who kept repeating the same words and was being ignored by the doctor and nurses. Then, the nurse in charge said, "Is this the patient assigned to your case report? This patient is my responsibility. You should no longer take care of this patient. (G2, P1).
Discriminatory treatment due to healthcare professionals' prejudice	Being Treated unequal when the training hospital is not affiliated with the students' school	• Limited educational opportunities when the training hospital is not affiliated with the student's school	For students whose training hospitals were affiliated with the school, they were allowed to observe procedures they could not perform. Although there were many procedures I had not seen, I was not given that opportunity because I attended a different school. (G2, P6)
			That nurse gives students from her own school an extra opportunity to observe. I became more cautious. (G3, P7)
		• Being treated differently by medical staff depending on the student's school affiliation	The nurse would bring snacks every day or every other day, but only for the students attending her alma mater. She also helped guide them with their case assignments. I thought those students were being treated better. (G2, P6)
			Among the many students, the nurse was particularly asking questions and encouraging only those from one specific school, which I found puzzling. Later, I realized that the nurse was an alumna of that school. (G3, P7)
	Being asked to practices based on gender stereotypes	• Nursing tasks that assigned to only male students	When tasks require physical strength, nurses often specifically seek out male students. (G3, P2)
			The nurse only gave presentation opportunities to the male nursing students. All the presentations were graded as well. However, the opportunities were distributed unfairly, which I felt was unjust. (G2, P6)
		• Male students' practical training being limited	Most of our trainees are female students. When a male student does something, it really stands out. If he is late or his vital signs are slightly slow, it is more noticeable than it would be with female students. Therefore, I think male nursing students face more restrictions. (G3, P7)
			male students were often prohibited from watching procedures such as Foley catheter insertions on female patients. However, when male patients received Foley catheters, all students observed together. (G3, P5)
